# The Roles of Gibberellins in Regulating Leaf Development

**DOI:** 10.3390/plants12061243

**Published:** 2023-03-09

**Authors:** Faujiah Nurhasanah Ritonga, Dandan Zhou, Yihui Zhang, Runxian Song, Cheng Li, Jingjuan Li, Jianwei Gao

**Affiliations:** 1 Shandong Branch of National Vegetable Improvement Center, Institute of Vegetables and Flowers, Shandong Academy of Agricultural Science, Jinan 250100, China; 2 Graduate School, Padjadjaran University, Bandung 40132, West Java, Indonesia; 3 College of Life Science, Shandong Normal University, Jinan 250100, China; 4 State Key Laboratory of Tree Genetics and Breeding, Northeast Forestry University, Harbin 150040, China

**Keywords:** bioactive, biosynthesis, crosstalk, metabolism, phytohormones

## Abstract

Plant growth and development are correlated with many aspects, including phytohormones, which have specific functions. However, the mechanism underlying the process has not been well elucidated. Gibberellins (GAs) play fundamental roles in almost every aspect of plant growth and development, including cell elongation, leaf expansion, leaf senescence, seed germination, and leafy head formation. The central genes involved in GA biosynthesis include GA20 oxidase genes (*GA20oxs*), *GA3oxs*, and *GA2oxs*, which correlate with bioactive GAs. The GA content and GA biosynthesis genes are affected by light, carbon availability, stresses, phytohormone crosstalk, and transcription factors (TFs) as well. However, GA is the main hormone associated with BR, ABA, SA, JA, cytokinin, and auxin, regulating a wide range of growth and developmental processes. DELLA proteins act as plant growth suppressors by inhibiting the elongation and proliferation of cells. GAs induce DELLA repressor protein degradation during the GA biosynthesis process to control several critical developmental processes by interacting with F-box, PIFS, ROS, SCLl3, and other proteins. Bioactive GA levels are inversely related to DELLA proteins, and a lack of DELLA function consequently activates GA responses. In this review, we summarized the diverse roles of GAs in plant development stages, with a focus on GA biosynthesis and signal transduction, to develop new insight and an understanding of the mechanisms underlying plant development.

## 1. Introduction

In the early 20th century, active compounds named gibberellic acids (GAs) were found and isolated from *Fusarium fujikuroi* [1]. In these few decades, GAs have also been identified in bacteria, fungi, algae, mosses, and viruses [2,3]. GA is a secondary metabolite product, a type of diterpene with a tetracyclic ent-gibberellin carbon skeletal structure, which is a natural complex biomolecule. Thus, GAs are phytohormones that are intensively involved in plant development [4]. Fundamental roles for GAs have been identified in almost every phase of plant development, including seed germination [5], flowering [6], leaf expansion [7], leaf formation [8], leaf angle [9], leaf size [10], and leaf senescence [11]. Since then, the signaling pathways, biosynthesis, catabolism, metabolism, distribution, and functions of GAs have been intensely observed, but still need to be specifically elucidated.

The GA biosynthesis pathway is significantly linked to GA biosynthetic enzymes [12], Ca^2+^ metabolism [13], and environmental factors [14]. The main GA-activating enzymes involved in GA biosynthesis are GA20 oxidases (*GA20oxs*) and GA3 oxidases (*GA3oxs*), while GA2 oxidases (*GA2oxs*) act as GA-inactivating enzymes [15,16,17]. The activity of *GA20oxs* (*GA20ox1*–*GA20ox5*) is crucial to determining the concentration of GA. An analysis of *GA20ox* transcripts revealed that their expression patterns differ, suggesting that they may play distinct roles in GA-regulated developmental processes [18]. Rieu et al. [18] revealed that *AtGA20ox1* and *AtGA20ox2* are most strongly expressed during vegetative and early reproductive development in Arabidopsis. It was also found that *GA20ox2* expression is tissue-specific or developmental stage-specific [19]. Similar to *GA20oxs*, *GA3oxs* also have a direct role in determining GA biosynthesis and bioactive GAs. In the GA biosynthetic pathway, a *GA3ox* catalyzes the last step, which results in the synthesis of bioactive GAs [20]. Meanwhile, *GA2ox* catalyzes bioactive and/or immediate precursors of GAs to form inactive GAs. The gene encoding *DkGA2ox1* is also well known as an important negative regulator of GA levels in plants or a dwarfism regulator gene, and it is a tissue-specific gene in *Diospyros kaki* [21]. In contrast, *AaGA2ox1*–*AaGA2ox3* transcripts were detected in all organs of *Artocarpus altilis*, but were mostly displayed in stems and leaves [22]. Following the previous study, bioactive GAs and GA biosynthesis gene expression varied among tissue organs, growth stages, and species [13,23].

More than 135 GAs have been chronologically defined, but their names are not given based on their position in the biosynthetic pathway [5]. The main bioactive GA is the early-13 hydroxylation pathway GA (GA_3_). Based on previous evidence, GA_3_ regulates organ formation and gravitropism by modulating the synthesis of the plant hormone auxin and transporters, and by increasing stress resistance [24]. In fact, the crosstalk between GAs and other phytohormones is complex and modulates plant growth and development [25,26,27,28,29]. Recent research has shown that GA_3_ is correlated with the enhancement of indole acetic acid (IAA) and zeatin (ZR), and to reduced abscisic acid (ABA). At the same time, the expression levels of genes related to hormone metabolism and signaling were substantially changed through a phytohormone analysis [30].

**Figure 1:**
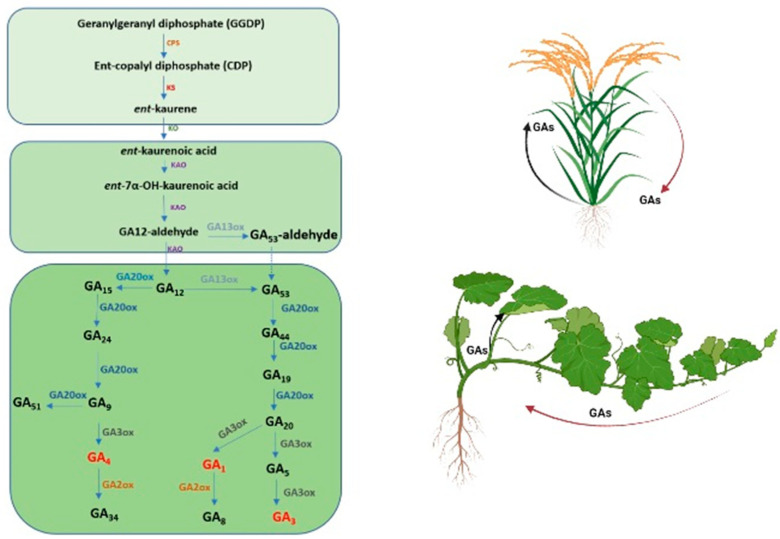
GA biosynthesis and endogenous bioactive GA levels in plants. Illustration of a schematic plant (**right**) and GA biosynthesis (**left**). The arrows are color-coded to correlate with GA forms shown in the biosynthetic pathway. Red arrows indicate the reduced level of GAs due to plant organ position, while black arrows indicate the increased level of GAs. In several plants, such as rice and watermelons, GA content is high at the base of the leaf or lower side [10,35]. In addition, several reports also found that bioactive GA content was reduced following the large length of the base to leaves [28]. In these cases, the exact level of GAs was not clear.

As shown in Figure 1, C20-GAs (GA_53-aldehyde_ and GA_12_) were used as the substrates during GA biosynthesis [31]. *GA20ox* catalyzes the conversion of GA_53_ to GA_20_ and GA_12_ to GA_9_ in three stages. Then, *GA3ox* catalyzes GA_20_ to GA_1_ and GA_9_ to GA_4_. In the last synthesis, *GA2ox* catalyzes GA_5_ to GA_3_ and GA_20_ to GA_1_ [32]. Briefly, lower GA concentrations were positively related to the reduced expression of GA biosynthetic genes and the transcriptional activation of GA catabolic genes [33]. GA metabolic pathways have also been extensively studied, and mutants with gene gains or losses in GA biosynthesis or catabolism change the GA levels in plants [17,34]. In dwarf *Zea mays*, it was found that the endogenous GA_1_ and GA_8_ levels in mutant plants were lower compared to the WT, while the concentration of endogenous GA_44_, GA_19_, GA_20_, GA_1_, and GA_8_ were higher compared to the WT in *GA20ox1*-overexpressing (OE) plants. Surprisingly, the transcript levels of *ZmGA20ox1*, *ZmGA3ox2*, and *ZmGA2ox* also increased in the *GA20ox1*-OE line. Altering GA levels significantly influences proportional changes in organ growth [28].

Previous studies found a correlation between the GA level and leaf distance from the base. As the length of the stem to the leaves increases, the bioactive GA level reduces. Normal cell elongation and differentiation in the stem are linked to leaf development with a higher bioactive GA content. GAs move from the leaves to the stem to provide sufficient bioactive GAs in the stem of rice [23]. In watermelons, GAs are high at the base of the leaf and virtually absent in the mature part [10]. In several cases, the asymmetric expression of GA-related genes was caused by gravity. The expression of *OsGA3ox1* on the lower side was higher (eight-fold) compared to the upper side in *Oryza sativa* [35] (Figure 1). In addition, bioactive GAs were not imported from other organs, but were locally synthesized [36].

GA biosynthesis and catabolism, as well as the physiological responses they cause, depend on many signals and many positive and negative transcriptional feedback mechanisms and feed-forward mechanisms. Understanding GA homeostasis at the molecular level may be enhanced by locating and controlling regulatory hubs, such as transcription factors (TFs), that regulate numerous enzymatic steps of GA metabolic pathways [25]. The investigation of TFs in regulating GA biosynthesis has also been reported [25,30]. For example, *HDZIP5/8* is correlated with *GA2ox*, and *HDZIP3/6* with *GID1*, to improve internode elongation [30], and *SGD2*/*OsHOX3* mediates GA biosynthesis by reducing the GA_1_ level in the sgd2 mutant [25]. Therefore, studies of the relationships among GA signal transduction, gene regulation, and GA modulation during various leaf development stages could elucidate the underlying molecular mechanisms and advance our understanding to reveal novel avenues for improving agricultural yields. This review focuses on regulating bioactive GAs, GA-related genes, GA biosynthesis, and the regulatory role of GAs in leaf development.

## 2. GA Level Affects Plant Phenotype

Based on cytological observations, a study found that a defective phenotype in a leaf was mainly caused by a decreased cell length caused by GA deficiency, which was controlled by the repression of GA biosynthesis, resulting in dwarfism [25]. However, the reduction in bioactive GA levels is a complex process. Light, carbon availability, and environmental factors affect the timing and magnitude of GA biosynthesis, resulting in the removal or enhancement of bioactive GA levels and downstream gene expression [37]. A reduction in leaf area, a thicker lamina, smaller abaxial pavement cells, a stomatal density enhancement, and a guard cell length reduction also presented in a GA-deficient sunflower [38]. In *O. sativa*, GA-deficient plants generally exhibit small grains, shorter panicles, a reduced leaf number, and a reduced internodal length [25]. Meanwhile, in Arabidopsis, short hypocotyls, delayed flowering, dwarfism, and male sterility are among the characteristics of GA-deficient plants [39].

GA deficiency can be solved by a GA_3_ application. A GA_3_ application results in an etiolated phenotype, including thinner leaves, a greater leaf area, and a reduced chlorophyll content [40]. In *Lactuca sativa* L. and *Eruca sativa* L., a GA_3_ application caused narrow and elongated leaves as well as lengthened internodes after 10–14 days of treatment [41]. In *Brassica oleracea* var. capitata, the head diameter and size of outer leaves increased in response to a GA_3_ application [42]. In *Camellia sinensis*, a GA_3_ application accelerated bud germination, but caused chlorophyll degradation resulting in photosynthesis reduction [43]. Meanwhile, GA_3_ and GA_4+7_ applied to the shoot apical meristems of cauliflower (*B. oleracea* var. botrytis) and broccoli (*B. oleracea* var. italica) caused earlier curd formation and triggered a vegetative-to-reproductive transition [44]. Based on a previous report, GA_3_ applications are more significant in GA-susceptible plants. For instance, vine elongation in dwarf pumpkins (GA-sensitive pumpkins) was more responsive to varying GA_3_ concentrations compared to the WT [45]. Apart from that, GA_3_ applications have a different role in each species. In watermelons, a GA_3_ application only increased shoot biomass [10], illustrating that each plant has intraspecific variation in its response to a GA_3_ application [46]. However, a GA_3_ application might cause an endogenous hormone imbalance via the regulation of hormone synthesis-related gene expression in *Physalis heterophylla* [47].

GA biosynthesis inhibitors such as paclobutrazol (PAC), uniconazole, and daminozide are used to observe the opposite effect of a GA application. PAC induced the growth of smaller plants with a dark green phenotype and increased leaf thickness in *Nicotiana tabacum* [48]. Similarly, daminozide showed the opposite effect compared to a GA application in *Stevia rebaudiana* [49]. A dense spongy parenchyma was found in PAC-treated plants [40]. Moreover, uniconazole strongly delayed the bolting time, stem elongation, and flowering time [14].

## 3. GAs Regulate Leaf Development

A drastic reduction in the GA level causes a huge disturbance at the metabolic level [50]. Metabolic pathway proteins are the main target of GA regulation, which function in signal transduction, metabolism, and defense processes [51]. GA-mediated signals are affected by GA receptors. GA receptors perceive GA and transmit signals to activate GA-regulated reactions and maintain GA-binding activity [7]. Due to the flexibility of leaf development, the leaf is referred to as a model for plant development [52]. The flexibility of morphogenesis and the differentiation of leaf development cause a huge diversity in leaf shape, leaf angles, leaf senescence, and leaf size [38,53,54]. However, flexibility is distinct in each plant species, and the environment as well as hormonal factors are crucial in leaf development [41,55,56]. During the various stages of leaf development, plant organs, stresses, GAs, and other phytohormones play important roles in regulating the transcription expression of downstream genes [57].

### 3.1. Leaf Size

Leaves are initiated at the flank of the shoot apical meristem (SAM). Cell division and expansion are critical processes in the formation of a complex, overlapping, and interconnected mature leaf [58]. Cell division and expansion play significant roles in determining cell number and size, respectively [26], and are correlated with cell volume, behavior, and other plant organs [59]. Cell proliferation occurs throughout the entire primordium during the first phase and generates new cells, the size of which is nearly stable and small. Cell proliferation can be improved with nuclear division and mitotic nuclear division [30]. When cell division in the developing leaves has ceased, further growth is primarily accomplished through cell expansion, leading to a significant increase in cell size in the second phase. The number of cells produced during the cell division phase of leaf development determines the final leaf size [58]. However, cell expansion contributes to differential leaf growth. The increased cell expansion is linked with an enhancement of the ploidy level, and the length of the cell expansion phase and the cell expansion rate can alter the final leaf area [60]. Apart from that, cell division and expansion are distinct between dicots and monocots. Cell division ceases earlier in the tip and continues longer in the base of the leaf in dicot plants. In monocot plants, cell division occurs earlier at the bottom of the leaf, then expands the cells, and subsequently matures the cells at the tip, illustrating that dicots and monocots have a temporary and spatial distribution of cell division and expansion [61]. Therefore, the temporospatial distribution of GAs may be quite different and determine the final leaf size.

GAs control leaf size by modulating cell division and increasing water absorption. Although cell size is not entirely related to leaf size or shape, the enhancement of protoplasmic content enlarges cell size, which is reflected in the surface area, diameter, and plant tissues [42]. Light, environmental factors, GAs, and DELLA proteins modulate the elongation rate of differentiated cells during the cell division phase. A longer photoperiod enhances GA biosynthesis, resulting in a higher expression of the key GA synthesis genes *GA20ox* and *GA3ox*, consequently inducing active GAs in Arabidopsis [62]. In contrast, a lower light availability reduced the expression of *GA20ox* and *GA3ox*, resulting in reduced leaf expansion [63,64] and demonstrating that *GA20ox* and *GA3ox* are dependent on the intensity and quality of light in *Rosa* sp. [37].

Meanwhile, cryptochromes and phytochromes were important in reducing the active GA content in visible light in *Solanum lycopersicum* [16]. Cryptochromes capture blue light and induce a robust expression of *OsGA2ox4-7*. In contrast, phytochrome B (phyB) captures red light with the auxiliary action of phyA to mediate *OsGA20ox2* and *OsGA20ox4* repression, resulting in a lower GA_1_ level, which leads to the suppression of leaf sheath elongation in rice. The regulatory mechanism of phyB in *O. sativa* is different from dicots. In dicots, a reduction in active GAs is mediated by phyA by the suppression of *PsGA3ox1* and the induction of *PsGA2ox2*. However, the phyA capacity could not decrease the active GA levels under B-light irradiation in peas [65]. Moreover, a blue-light inhibitor of cryptochrome 1 and 2 (*BIC1* and *BIC2*) is cryptochrome-interacting proteins. It functions in repressing cryptochrome *2 (CYR2*) activity by participating in the GA regulation pathway by regulating the endogenous bioactive levels of GAs [66]. Then, GA biosynthesis and the metabolic gene transcript level were mediated by active GAs, suggesting that *OsBIC1* and *OsCRY1s* are involved in GA pathway regulation [66]. In *Glycine max*, the upregulation of *GmGA2ox* genes was mediated by *GmCRY1*, but repressed by low blue light (LBL), suggesting the involvement of GA homeostasis in LBL-induced cell elongation [67].

**Figure 2:**
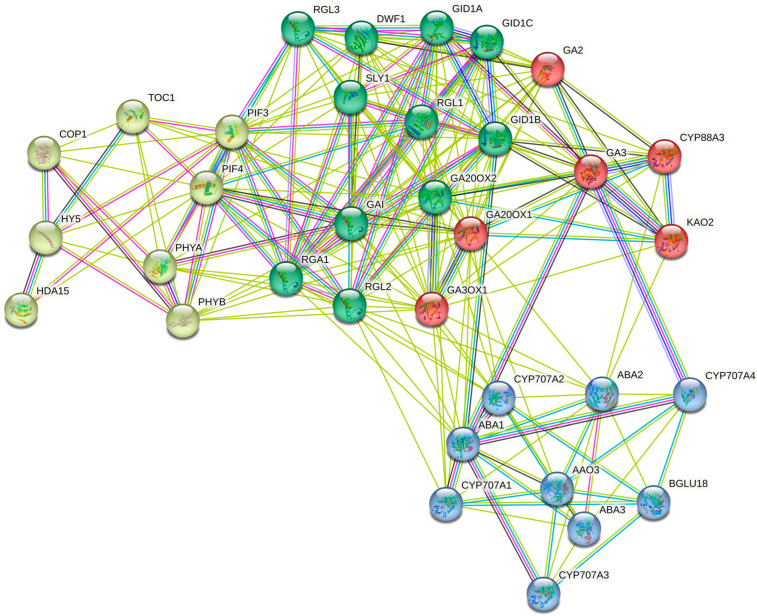
Interaction network analysis of all genes related to GA biosynthesis identified in *A. thaliana* using STRING. The interaction network had significantly more interactions than expected. A total of 34 *A. thaliana* proteins are involved in more interactions, indicating that the proteins are at least partially biologically connected as a group. The protein cluster color is related to the type of proteins. The yellow color relates to GA biosynthesis light regulation, the green color relates to DELLA proteins, the red color relates to GA biosynthesis proteins, and the blue color relates to phytohormones. The line color is related to the type of interaction. The green line shows the gene neighborhood, the pink line means experimentally determined, the red line means gene fusion, the black line means co-expression, the dark blue line means gene co-occurrence, and the blue line represents protein homology. (For more interpretations of the color codes in this figure legend, the reader is referred to the web version of this network analysis https://string-db.org/cgi/network?taskId=bYb8HEacei7t&sessionId=bUXWyy80zCr5 accessed on 2 March 2023).

The COP1/HY5 pathway mediates GA biosynthesis light regulation, while GAs modulate light signaling to prevent de-etiolation in the darkness. As mentioned previously, *CRY1* is a light receptor that functions in repressing GA signaling by interacting with DELLA proteins (GAI, RGA, RGL1, RGL2, and RGL3) and *GID1* to inhibit the GID1–GAI interaction competitively. *TaCRY1a* interacts with *TaGID1* and *Rht1* to minimize the interaction of *TaGID1* and *Rht1* in *Triticum aestivum* transgenic lines [68]. DELLA proteins act as plant growth suppressors by inhibiting the elongation and proliferation of cells. In addition to repressing GA responses, DELLA proteins act as a central node to integrate light and temperature inputs and other hormone signaling pathways. Bioactive GA levels are inversely related to DELLA proteins, and a lack of DELLA function consequently activates GA responses [39]. As shown in Figure 2, protein interactions are complicated and inter-connected. We used the STRING tool (https://version-11-5.string-db.org/, 10 February 2023) to identify the protein–protein interaction networks involved in GA biosynthesis, responses, perceptions, and signal transduction pathways in Arabidopsis. The k-means clustering was chosen as the clustering option for four cluster proteins: red, yellow, green, and blue. As shown in Figure 2, the GA–GID–DELLA interaction is complex and relates to other cluster proteins such as *HY5* and *ABA2*, illustrating that each protein relates to and affects each other.

Most consistently, GA-regulated genes contain auxin and BR-related genes [39,48]. The GA signaling pathway is a fast pathway, as shown by significant changes in the expression levels of GAs after GA disruption [48]. *ZmGA2ox3* and *ZmGA2ox10* expression were increased by ethephon, which acts as a plant growth regulator, and at the same time, *ZmPIN1a*, *ZmPIN4*, and *ZmGA3ox1* transcript levels were reduced, resulting in decreased auxin and GA accumulation in *Z. mays* [69]. A previous study also reported that exogenous BR induces GA biosynthesis genes as well as auxin biosynthesis, which can be seen by the rapid responses of several BR, auxin, and GA-related genes to the BR and BR inhibitor treatments expected to result in BR, IAA, and GA crosstalks [24]. However, BR and GA have different roles in promoting leaf size independently. A study revealed that mixing BR with GA biosynthesis inhibitors showed a synergistic effect in the retardation of rice seedlings compared to non-mixed BR and GA biosynthesis inhibitors [70]. BR signaling gene expression can be inhibited by the SLENDER RICE1 (SLR1) protein in response to GA induction [71], while JA requires SLR1-mediated GA pathway suppression [72]. According to a previous study, jasmonate (JA) signaling antagonized GA biosynthesis in Arabidopsis [73]. In *Pharbitis nil*, GAs repressed endogenous JA and JA biosynthesis by collaborating with an inhibitor of JA biosynthesis, salicylhydroxamic acid [74]. In Arabidopsis, it was revealed that a lack of ABA tends to dominate leaf growth, regardless of GA levels. The involvement of genes related to leaf development was found in the ABA–GA interaction, suggesting that the ABA and GA interaction has a genetic relationship. To regulate leaf growth, multiple coordinated pathways are used rather than a single linear pathway [75].

*CsGA20ox1* was positively correlated with cytochrome P450 90A1 (*CsCPD*) and Dwarf4 (*CsDWF4*) in response to BR biosynthesis [76]. GA and BR promoted the homeobox TF, *HB40*, induced by GA and, in turn, decreased the levels of endogenous bioactive GAs by inhibiting GA biosynthesis while increasing the GA deactivation gene. HB40 directly activates the transcription of JUNGBRUNNEN1 (*JUB1*), a key TF that restricts growth by impairing GA biosynthesis and signaling. *HB40* also induces genes that encode *GA2oxs*. Nuclear growth-repressing DELLA proteins induced HB40 in plant growth [39]. The actualization of GA interactions with other phytohormones can also be seen in *Nelumbo nucifera* in the promotion of leaf sheaths [77].

**Figure 3:**
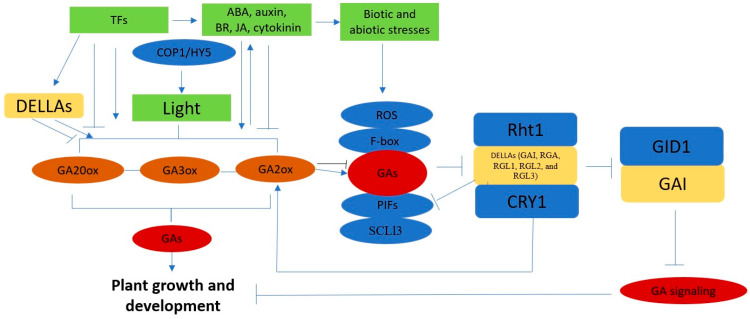
GA biosynthesis mechanism and inactivation of GA signaling. GA signaling was influenced by DELLAs, GID1, and GAs. Light, TFs, biotic and abiotic stresses, and COP1/HY5 affected GA binding to ROS, F-box, PIFs, and SCL13 to degrade DELLAs. CRY1 repressed GA signaling by binding with DELLA proteins (GAI, RGA, RGL1, RGL2, and RGL3) and GID1 to inhibit the GID1–GAI interaction. The green boxes indicate other factors that affect GA biosynthesis, the blue and yellow boxes indicate other genes that affect or are affected by GAs, the brown circles relate to GAs activating and inactivating genes, and the red boxes indicate bioactive GAs.

As previously mentioned, TFs regulate leaf size and might positively and negatively correlate with GAs [78]. A dehydration-responsive element-binding protein (*SlDREB*) from *Solanum lycopersicum* was mainly expressed in the leaf suppressed by GAs, but induced by ABA. In a *SlDREB*-OE plant, the expression of the key genes ent-copalyl diphosphate synthase (*SlCPS*), *SlGA20ox1*, *SlGA20ox2*, and *SlGA20ox4*, which are involved in GA biosynthesis, were downregulated, causing lower endogenous bioactive GA and resulting in a decline in leaf expansion and internode elongation. In *GA20ox1*-OE lines, the bioactive GA level increased, leading to the production of more and larger cells and resulting in larger leaves [79]. Meanwhile, the increased levels of GA could result in a simplified leaf form [80]. Contrastingly, abiotic stresses minimized the cell flux and elemental growth rate of leaves by degrading GA synthesis, causing a reduction in *GA20ox4* [10]. In addition, extended morphogenetic activity was positively correlated with a compound leaf shape. The CIN-TCP TF LANCEOLATE (LA), which promotes leaf differentiation, was negatively regulated by miR319 and mediated in part by GA, which can be elucidated from the expression of *SlGA20ox1* and *SlGA2ox4* [80]. GA also induced other TFs during leaf elongation, such as bHLH [81] and HD-ZIP II [78], resulting in the suppression of GA biosynthesis and signaling due to the higher expression of these TFs. In addition, nonenzymatic cell wall proteins such as α-expansins were also involved in GA stimulation during leaf elongation [82]. In conclusion, bioactive GAs, GA biosynthesis genes, DELLAs, light, TFs, phytohormone crosstalk, and other proteins were involved in regulating leaf size by affecting the cell size and number and, eventually, the size of the leaf (Figure 3).

### 3.2. Leaf Angle

The leaf angle refers to the angle formed by the stem and leaf blade’s adaxial side, which forms the plant architecture into a compact or broad structure. It affects the competition and interception of light among plants, resulting in increases or decreases in plant yield and productivity. The leaf angle is an important agronomic trait due to its effect on sunlight capture efficiency and nitrogen reservoirs [83]. Several species can actively adjust their leaf angles to minimize the detrimental effects of abiotic stresses. Plants may take advantage of an environmental resource, such as light or oxygen, or avoid environmental stresses [84]. Plants maximize carbon gain through homogenizing the light distribution caused by vertical leaf orientation, resulting in greater light capture for photosynthesis. In addition, vertical leaf orientation could reduce the radiation load at mid-day and be related to stress avoidance. Apart from that, plants have a particular type of leaf movement called hyponastic growth, known as an upward re-orientation of rosette leaves [85]. Hyponastic growth is initiated by cell differentiation, cell elongation direction, or cell proliferation and is also affected by biotic stresses such as submergence, heat, or even shade [86,87]. Due to the role of GAs in cell elongation, we assumed that GAs contribute to the leaf angle.

ABA and GA have different roles in regulating leaf angles. ABA acts as a negative regulator of petiole elongation [88]. As previously observed, ABA levels were reduced in an ethylene-dependent manner in *Rumex palustris* by an increase in ABA catabolism, but inhibition of ABA biosynthesis. The correlation of ABA and GA occurs during submergence conditions where ABA mediates GA production for elongation [89]. In a previous study, the GA level was incompatible with inducing hyponastic growth, implying that the response was saturated by endogenous GA levels [85].

Furthermore, it has also been revealed that specific GA-related genes interact with BR genes to regulate the leaf angle [9,83]. *OsOFP22* interacts with 3-aa loop extension (TALE) homeodomain proteins and BR signaling components to regulate BR/GA responses and biosynthesis [71]. Two GA signaling pathways regulate the leaf angle through crosstalk with BR signaling, namely the GA-GID1-DELLA pathway and the G-protein-dependent pathway. GA signaling was repressed by the absence of GAs, *SLR1*, and DELLA proteins [90]. When GA binds to GID1 and strengthens the interaction in both *GID1* and DELLA, DELLA is rapidly degraded through the ubiquitin–proteasome pathway, causing GA signaling repression. On the other hand, in the G-protein-dependent pathway, D1/RGA1, a subunit of the G-protein (Ga), plays a crucial role in the leaf angle. GA binds to an extracellular receptor, signals are transduced to effector proteins by Ga proteins, and finally, the suppressive activity of *SLR1* was eliminated [91]. *OsMIR396d* was also found to be involved in GA and BR signaling and the regulation of internal GA biosynthesis; consequently, GA signaling pathway gene (*OsGID2*) and GA biosynthesis gene (*OsCPS1*, *OsKO2*, *OsGA20ox1*, and *OsGA20ox3*) transcript levels decreased. In contrast, BR signaling was enhanced in *OsMIR396d*-OE plants, implying that *OsmiR396d* could control the leaf angle through GA and BR signaling [92].

### 3.3. Leafy Head Formation

The leafy head is a unique organ in vegetables and is used as an identifying trait in the Brassicaceae family, which includes plants such as Chinese cabbage (*Brassica rapa* L. ssp. *Pekinensis*). Chinese cabbage has four growth stages: seedling, rosette, folding, and heading. The leaves grow upward and inward after the rosette stage, and the leafy head forms when the leaf whorls intertwine [93]. The size, shape, density, and uniformity of leafy head formation significantly influence the commercial value [94]. As a complex trait, the development of the leafy head includes incurve and leaf blade enlargement, the shortening of the petiole, or a change in the leaf angle that is correlated with petiole morphology [95].

At the heading stage, the leaf morphology of the inner and outer leaves differs. The inner leaves are insulated from light at this stage and serve as storage organs. On the other hand, the outer leaves are green and exposed to light, implying that they can photosynthesize and provide a carbon source for head development [96]. The combination of high temperatures and elevated CO_2_ induces severe leaf hyponasty/angles in tomatoes, which is assumed to be correlated with a leafy head [97]. Uneven auxin distribution is critical in leafy head formation due to its relationship to polar auxin transport [8,98]. In addition, three families of auxin transport genes have been discovered to be expressed throughout the entire process of leafy head formation: auxin-resistant 1/like aux1 (AUX/LAX), pin-formed (PIN), and ATP-binding cassette subfamily B (ABCB/MDR/PGP), suggesting that these genes play important roles in the development of heads [8]. Besides dynamic auxin distribution, low temperatures, wide temperature differences, low light intensity, short light duration, and sufficient carbon, GA promotes leafy head induction and development [99]. The mechanism of GA in forming leafy heads is complex and still needs to be explored.

Previous studies have revealed that GA affects leafy head formation in Chinese cabbage [34,93]. However, the role of GA in regulating leafy head formation in a plant is still limited and needs to be elucidated. *BrKS1*, *BrCPS1*, and *BrKAO2* play important roles in GA biosynthesis to regulate leafy heads. A mutation of these genes caused a non-heading mutant (nhm) in *B. rapa*. *BrKS1* encodes an ent-kaurene synthase, the key enzyme involved in GA biosynthesis. Ent-kaurene synthase functions as a catalyst in the cyclization or rearrangement of the substrate ent-copalyl diphosphate (ent-CPP) to produce ent-kaurene, a precursor of GAs [34]. Similarly, *BrCPS1*, which encodes ent-CPP synthase 1, was also discovered to be involved in GA biosynthesis. A mutation in the fourth and seventh exons of *BrCPS1* was responsible for nhm phenotypes. The GA content of mutant plants was lower than for the WT, and the tendency to form leafy heads was found after a GA_3_ application in nhm lines [93]. MutMap and kompetitive allele-specific PCR genotyping identified *BrKAO2* as the candidate gene, which encodes ent-kaurenoic acid oxidase 2 in the GA biosynthetic pathway. Two non-synonymous mutations in the second *BrKAO2* exon caused mutations named nhm3-1 and nhm3-2. *BrKAO2* was expressed at all stages of leaf development [17]. A list of representative cloned genes related to GA biosynthesis and their functions is presented in Table 1.

Although the expression of auxin-regulated genes was dominant in *B. rapa* for leafy head formation, a total of ten GA-related genes (*BrGASAs*, *BrGA2oxs*, and *BrGA20oxs*) were increased [100]. Moreover, auxin synthesis genes incited the formation of adventitious roots to increase the root number and improve the character of leaf formation in *B. rapa* [96]. In another study, *LsKN1* played an important role in leafy head development by binding to the promoter of *LsAS1* to alter the dorsoventral pattern. However, the increased *LsKN1* expression was prominent, but deficient for heading compared to GA biosynthesis genes [102]. *LsKN1* promoted auxin biosynthesis and repressed the biosynthesis of GA in lettuce (*L. serriola*). *LsKN1* triggered the expression of two critical genes in the GA biosynthesis pathway (*LsGA3ox1* and *LsGA20ox1*) by binding to their promoter regions [101]. Similar to *LsKN1*, the transcription coactivator ANGUSTIFOLIA (*BrAN*) from *B. rapa* also regulated leafy head formation by mediating the leaf width and regulating the arrangement of cortical microtubules and the shape of pavement cells [107].

Interestingly, the *BrAN3* level was different in the rosette and heading stage. *BrAN3* RNAi lines showed changes in phytohormone signaling pathways, including auxin, ethylene, GA, JA, ABA, BR, CK, and SA. Two DEGs (Bra039460 and Bra003520) related to GA signaling pathways were downregulated in *BrAN3* silencing plants, contributing to leafy head formation and proving to be positively correlated with *BrAN3* [108]. However, several lines of evidence showed that the rosette stage plays a major role in leafy head formation, i.e., the higher expression of *BrAN3* in rosette leaves affects leafy head formation. This assumption was reinforced by Alemán-Báez et al. [103], who found that rosette leaves are the largest leaves of *B. oleracea* and provide the majority of the energy required to produce the leafy head. BolC02g009950.2J and Bol035882, similar to OFP8, influenced the leaf ratio by regulating cell elongation and by repressing *GA20ox1* at the rosette and heading stages. Other candidate genes that may control leafy head formation are *BrpSAW1*, *BrpESR1*, and *BrpGL1*, which are also related to trichomes, petioles, serration, and cell division in *B. rapa* [109].

Moreover, prohexadione-calcium (Pro-Ca) contributes to leafy head development in *B. rapa* and *Chrysanthemum morifolium* by reducing or increasing endogenous bioactive GAs, indicating that GA biosynthesis is inactivated/activated by Pro-Ca [110,111]. In *A. graveolens*, 12 lignin synthesis gene expression profiles changed with an increase in the GA concentration and GA biosynthesis genes, including *AgKO*, *AgKAO*, *AgGA20ox1*, *AgGA20ox2*, *AgGA3ox1*, *AgGA2ox1*, and *AgGA2ox3*. Contrastingly, the *AgKS* and *AgGA2ox2* expression was reduced with an increase and change in the GA biosynthesis genes and bioactive GAs as well as the lignin synthesis gene, demonstrating that lignin might be correlated with leafy head formation [105,112]. The increased synthesis activity of *GA20ox1* with the reduced inactivation activity of *GA2ox1* to encode enzymes resulted in the final level of bioactive GAs, including GA_1_ and GA_4._ GA_1_ and GA_4_ levels are prominent factors leading to cellulose biosynthesis, resulting in reduced lignin content in *Sorghum bicolor* [23]. In fact, the transcript levels of *GA20ox1* were not correlated with the levels of bioactive GA in Arabidopsis [104]. In summary, how leafy head mechanisms function together through GA and phytohormone crosstalk, as well as the involvement of any other factors, needs to be studied in greater detail.

### 3.4. Leaf Senescence

Leaf senescence is the final stage of leaf development, significantly influencing plant survival and productivity [26]. Senescence is distinguished by color changes as well as a functional transition from nutrient absorption to nutrient remobilization and is regulated by age, phytohormones, and environmental stresses [113]. Plants have two types of senescence: mitotic and post-mitotic [114]. Mitotic senescence occurs in SAM, whereas organs such as leaves and flowers conduct post-mitotic senescence [115]. When senescenced leaves are degraded, their nutrients are transferred to other developing organs, such as new buds, young leaves, flowers, and seeds, resulting in the death of the senescing leaves [113].

Plant hormones are a major player affecting leaf senescence during the initial development, progression, and terminal phase of senescence. GA, cytokinin, and auxin delay leaf senescence [116,117], while ethylene, JA, SA, and ABA promote leaf senescence [26,118,119,120]. It should be noted that the characterized functions of each hormone in different plant species or plants with different mutation backgrounds is different due to their complicated crosstalk [113]. On the other side, a GA inhibitor application promotes leaf senescence, consequently reducing the bioactive GA content. Previously, it was found that GA does not directly influence leaf senescence, but can delay it by antagonizing ABA [121].

Based on a previous study, GA biosynthesis genes, GA deactivation genes, respiratory burst oxidase homolog genes (*RBOHs*), senescence-associated genes (*SAGs*), and chlorophyll catabolic genes (*CCGs*) increased due to the application of exogenous ABA. The increased level of endogenous ABA caused a reduction in the GA_3_ level, resulting in accelerated leaf senescence, possibly by the simultaneous suppression of gene expression associated with ABA catabolism and GA biosynthesis [118]. Contrastingly, the endogenous GA level was affected by an exogenous GA treatment by the regulation of the genes encoding the GA metabolic pathway. Among the identified structural genes involved with GA_3_-inhibited senescence, a GA_3_ treatment inhibited *RBOHs*, *SAGs*, *CCGs*, and GA deactivation genes, whereas the GA biosynthesis genes were induced in GA_3_-treated cabbages [122].

In contrast to GA, dark and cold conditions induced rapid leaf senescence [55,123,124]. However, the combination of non-13 hydroxylation (GA_4+7_) and light supplementation could prevent rapid leaf senescence caused by dark and cold conditions in *Lilium* sp. A drastic reduction in photosynthetic pigments and antioxidant activity, proteolysis, and lipid peroxidation enhancement can be prevented by both light and additional GA_4+7_ [125]. Furthermore, since the degradation of photosynthetic pigments is one of the causal factors of leaf senescence, any agent’s effect on chlorophyll degradation is directly related to leaf senescence.

The relationship between DELLA and GA in leaf senescence has been identified. Although DELLA is a GA repressor, DELLA contributes to reducing leaf senescence in plants. A study investigated a GA-deficient mutant (*ga1-3*) and a Q-DELLA/*ga1-3* mutant whose DELLA repressor was removed to evaluate the relationship between DELLA and GA in Arabidopsis leaf senescence. The transcript levels of senescence-specific genes (*SAG12* and *SAG29*) in the leaves of the WT were much lower compared to those of the Q-DELLA/*ga1-3* mutant during senescence, and vice versa with the *ga1-3* mutant, illustrating that leaf senescence is influenced by the removal of the DELLA repressor [11].

A close relationship exists between GA signaling and TFs. However, upstream TFs that control GA biosynthesis in association with GA-mediated leaf senescence remain unclear. A member of NAC TF, NAP/ANAC029 (NAC-like, activated by APETALA 3/PISTILLATA), was found to be involved in leaf senescence in Arabidopsis. NAP positively regulated age-dependent and dark-induced leaf senescence by inducing senescence-related gene (*SAG113* and *AAO3*) expression related to chlorophyll degradation. The RGA and GAI interaction with NAP impaired the transcriptional activities of NAP on *SAG113* and *AAO3* expression, which was related to chlorophyll degradation, illustrating that NAP plays a considerable role in GA signaling [119]. Fan et al. [122] stated that *BrNAC08* regulates leaf senescence by modulating structural genes (*BrPPH*, *BrRCCR*, and *BrGA2ox1*) in *B. rapa*. NAC binding sites (NACBS) were found in the promoter regions of those structural genes and were specific [122]. Meanwhile, ABA regulates *BrNAC041* at the transcriptional and translational levels, but contributes to GA biosynthesis to regulate leaf senescence. The transcription patterns of *BrNAC041* were opposite from those of *BrCYP707A3*, *BrKAO2*, and *BrGA20ox2* and acted as transcriptional repressors to regulate the gene functions of *BrCYP707A3*, *BrKAO2*, and *BrGA20ox2*. *BrNAC41* binds to the NACBS of *BrCYP707A3*, *BrKAO2*, and *BrGA20ox2* promoters and was validated using an electrophoretic mobility shift assay (EMSA) and a chromatin immunoprecipitation (ChIP)-qPCR assay [118].

Although recently discovered, WRKY TF is becoming an important TF in leaf senescence. *BrWRKY6* is positively correlated with leaf senescence in *B. rapa*. *BrWRKY6* suppresses *BrKAO2* and *BrGA20ox2* while activating *BrSAG12*, *BrNYC1*, and *BrSGR1* by binding to their promoters via the W-box cis-element. In contrast, an exogenous GA_3_ application completely repressed leaf senescence, maintained a greater maximum quantum yield (Fv/Fm) and chlorophyll content, reduced electrolyte leakage and the expression level of a series of SAGs and CCGs, and increased GA biosynthetic gene transcript levels [126]. Previous research has shown that *WRKY75* also contributes to leaf senescence. An involvement of *AtWRKY75* was found in regulating age-triggered leaf senescence through a GA pathway in Arabidopsis. *WRKY75* regulates leaf senescence through the GA-mediated signaling pathway, illustrating that *WRKY75* is a part of the GA-mediated senescence regulatory network. GA-treated plants showed an enhancement of photosynthetic pigments and *SAG12*, demonstrating that the leaf senescence caused by GA was delayed in wrky75 mutants, but was accelerated in *WRKY75*-OE plants [127]. In the current study, *WRKY45* was a positive regulator of age-dependent leaf senescence. *WRKY45* mutations led to increased leaf longevity, whereas *WRKY45*-OE plants remarkably increased age-triggered leaf senescence. Senescence-associated gene (*SAG12*, *SAG13*, *SAG113*, and *SEN4*) transcript levels were significantly increased in *WRKY45*-OE plants. *WRKY45* binds to the promoter of senescence-associated genes and interacts with RGA-LIKE1 (*RGL1*), a GA signaling pathway repressor [128].

A TEOSINTE BRANCHED1/CYCLOIDEA/PCF (TCP) TF, *BrTCP21*, was involved in GA-delayed leaf senescence. A GA_3_ application maintained a higher expression of *BrTCP21* and suppressed the expression of senescence-associated genes by binding to the promoter region of *BrGA20ox3* to activate its transcription, thus delaying leaf senescence. In senescing leaves, the *BrTCP21* transcript level was reduced and gradually decreased following leaf senescence [15]. However, the relationship among GAs, other hormones, and TFs with regard to leaf senescence can be complicated. Thus, more experiments are needed to elucidate the mechanism. A list of TFs correlated with GA biosynthesis is shown in Table 2.

## 4. Conclusions and Future Perspectives

Studies about phytohormones have become increasingly important and helpful in finding a way to increase the yield and productivity of crops. In this review, we summarized the roles of GAs in plant organs and developmental stages. Bioactive GAs and GA biosynthesis gene expression varied among plant development stages and species. The main genes involved in GA biosynthesis are *GA20oxs*, *GA3oxs*, and *GA2oxs*, and these genes are correlated with bioactive GAs. The roles of GAs in regulating leaf size, leaf angle, leafy head formation, and leaf senescence have been revealed to correlate with other phytohormones, such as auxin, BR, SA, ABA, and JA. However, the mechanism of GAs in regulating leaf development is a complicated process that is still not fully understood, especially for leafy head formation. As mentioned previously, numerous factors in GA biosynthesis, namely light, Ca^2+^ metabolism, water availability, environmental stresses, various phytohormone interactions, varied species, and growth stages, remain to be determined [67,84,118]. Understanding the relationships among those aspects will provide new insights and understanding into leaf development studies. Thus, more focused studies on the GA biosynthesis mechanisms are needed to improve our understanding of leaf development.

## Figures and Tables

**Table 1:** Representative cloned genes associated with plant development related to GA biosynthesis.

No	GA Biosynthesis Genes	Species	Function	Reference
1	*OsGA2ox6*	*O. sativa*	Dwarfism, flowering, and seed production	[31]
2	*ZmGA20ox1*	*Z. mays*	Reduces dwarfism	[28]
3	*RoGA20ox*, *RoGA3ox*, and *RoGA2ox*	*R. rubiginosa*	Branching pattern and bud burst	[37]
4	*AaGA2ox1*–*AaGA2ox3*	*A. altilis*	Internode elongation	[22]
5	*OsCPS1*, *OsKO2*, *OsGA20ox1*, and *OsGA20ox3*	*O. sativa*	Leaf angle	[92]
6	*BrKS1*	*B. rapa*	Leafy head	[34]
7	*BrCPS1*	*B. rapa*	Leafy head	[93]
8	*BrGASAs*, *BrGA2oxs*, and *BrGA20oxs*	*B. rapa*	Leafy head	[100]
9	*LsGA3ox1* and *LsGA20ox1*	*Lactuca serriola*	Leafy head	[101,102]
10	*BolGA20ox1*	*B. oleracea*	Leafy head	[103]
11	*SbGA2ox1*	*Sorghum bicolor*	Stem biomass	[23]
12	*GA20ox1*	*Arabidopsis*	Leaf size and shape	[104]
13	*AgKO*, *AgKAO*, *AgGA20ox1*, *AgGA20ox2*, *AgGA3ox1*, *AgGA2ox1*, *AgGA2ox3*, *AgKS*, and *AgGA2ox2*	*Apium graveolens*	Leafy head	[105]
14	*NtGA20ox1*, *NtGA20ox2*, *NtGA3ox1*, and *NtGA2ox1*	*N. tabacum*	Bolting and flowering	[36]
15	*GA20ox4*	*Z. mays*	Elongation zone	[10]
16	*GA20ox2*	*Citrus unshiu*	Fruit set	[19]
17	*ZmGA2ox3*, *ZmGA2ox10*, and *ZmGA3ox1*	*Z. mays*	Promote fruit ripening, abscission, and flower induction	[69]
18	*VpGA20ox* and *VpGA3ox*	*Viola philippica*	Flower development	[62]
19	*CsGA20ox1*	*C. sinensis*	All development stages	[76]
20	*OsGA20ox2*	*O. sativa*	Internode	[90]
21	*OsKAO*, *OsGA3ox2*, and *OsGA2ox2*	*O. sativa*	Semi-dwarf	[106]
22	*BrKAO2*	*B. rapa*	Leafy head	[17]

**Table 2:** TFs related to GA biosynthesis in plants.

No	TFs	TF Family	GA Biosynthesis Genes	Species	Function	Reference
1	*HDZIP5*, *HDZIP8*, *HDZIP3*, and *HDZIP6*	HD-ZIP	*GA2ox GID1*	*C. purpureus*	Improve internode elongation	[30]
2	*SGD2/OsHOX3*	HD-ZIP		*O. sativa*	Improve height and grain size	[25]
3	*SlDREB*	AP2/ERF	*SlGA20ox1*, *SlGA20ox2*, and *SlGA20ox4*	*S. lycopersicum*	Leaf expansion and internode elongation	[16]
4	*LANCEOLATE (LA)*	CIN-TCP	*SlGA20ox1* and *SlGA2ox4*	*S. lycopersicum*	Leaf differentiation	[80]
5		bHLH		*O. sativa*	Leaf sheath elongation	[81]
6	*HAT1*	HD-ZIP II	DELLAs (*GAI* and *RGA*)	*Arabidopsis*	Enlarges cotyledons and trichome initiation	[78]
7	*BrNAC08*	NAC	*BrPPH*, *BrRCCR*, and *BrGA2ox1*	*B. rapa*	Leaf senescence	[122]
8	*BrWRKY6*	WRKY	*BrKAO2* and *BrGA20ox2*	*B. rapa*	Leaf senescence	[126]
9	*WRKY75*	WRKY	*SAG12*	*Arabidopsis*	Leaf senescence	[127]
10	*BrTCP21*	TCP	*BrGA20ox3*	*B. rapa*	Leaf senescence	[15]
